# Metagenomic sequencing discloses the virome composition of mosquitoes and sandflies from Central-Southern Tuscany, Italy

**DOI:** 10.1128/spectrum.01867-25

**Published:** 2026-02-26

**Authors:** Gianni Gori Savellini, Giulia Alessandri, Giada Beligni, Davide Badano, Pietro Paolo Fanciulli, Francesco Frati, Maria Grazia Cusi

**Affiliations:** 1 Department of Medical Biotechnologies, University of Siena 9313, Siena, Italy; 2 Department of Life sciences, University of Siena 165399, Siena, Italy; Universidade Federal do Rio de Janeiro, Rio de Janeiro, Brazil

**Keywords:** metagenomic, arboviruses, virome, phlebovirus, flavivirus

## Abstract

Mosquitoes and sandfly species are well-known vectors of viral pathogens of public health concern. However, the diversity and ecology of viruses within mosquitoes, including those responsible for clinical and sub-clinical infections in humans, remain poorly understood. In this study, we investigated the presence of phleboviruses and flaviviruses in *Aedes albopictus* and *Culex pipiens* mosquitoes, as well as Phlebotominae species, collected from the Siena and Grosseto districts (Tuscany, Italy) during the 2022–2024 summer season. Furthermore, *A. albopictus* and *C. pipiens* larvae were reared under laboratory conditions, and adults were collected for further virological analysis. Molecular investigations (reverse-transcription polymerase chain reaction [RT-PCR]) detected phleboviruses and/or flaviviruses in several batches of both field-collected and laboratory-reared flies. Notably, the highest incidence and co-circulation of both viral genera were observed in samples from the 2024 season. Furthermore, metagenomic analysis was performed on only 42 out of 67 RT-PCR-positive pools of mosquitoes and sandflies. This approach aimed to identify wild-type or recombinant viruses and assess the virome of autochthonous arthropods, contributing to knowledge on viral ecology in southern Tuscany and potential threats to humans. The resulting data revealed a wide viral community shared among mosquitoes, spanning over 30 taxonomic virus families, albeit no potential human pathogen virus was identified. Furthermore, our findings confirmed the mosquito specificity of certain endogenous arthropod viruses and provided evidence of their potential transovarial transmission in some cases. The present study provides a comprehensive analysis of the mosquitoes and sandflies virome, contributing to viral surveillance efforts and underscoring the need for enhanced monitoring of arthropod-borne pathogens.

**IMPORTANCE:**

In this study, we analyzed the co-circulating phleboviruses and flaviviruses, providing foundational data on the diversity, composition, and transmission of insect-specific and vector-borne viruses in Central-Southern Tuscany, an area increasingly exposed to arbovirus threats due to climate change and globalization. This is the first comprehensive metagenomic study to characterize the virome of *Aedes albopictus*, *Culex pipiens*, and *Phlebotomine* spp. in this region. Furthermore, we identified for the first time Punique virus (PUNV) in Italy, a phlebovirus with potential (though not yet confirmed) human pathogenicity.

## INTRODUCTION

Mosquitoes (Diptera: Culicidae) are well-known vectors of viral pathogens, transmitting diseases that impact millions of people worldwide (1–3). Arthropod-borne viruses (arboviruses) pose a significant global public health threat, primarily spreading through the bites of infected mosquitoes of the *Aedes*, *Anopheles*, and *Culex* genera (2, 4–6). Dengue virus (DENV), Zika virus, Chikungunya virus, Yellow fever virus, and West Nile virus (WNV) are among the most prevalent arboviruses. These pathogens are responsible for a wide range of clinical manifestations, from mild febrile illness to severe neurological and hemorrhagic diseases (7, 8).

The distribution of arboviruses is influenced by several ecological, climatic, and socio-economic factors. Indeed, urbanization, globalization, and climate change have contributed to both the diffusion of mosquito genera in regions where their presence was previously absent and the introduction of non-autochthonous species in geographical areas (9–12). Thus, the incidence of arboviral outbreaks dramatically increases worldwide year by year (4, 9–12).

Most of the known arboviruses, such as those belonging to the *Flaviviridae*, *Togaviridae*, *Peribunyaviridae,* and *Phenuiviridae* families, are RNA viruses that possess greater genetic diversity compared to DNA viruses, due to the error-prone nature of the viral RNA-dependent RNA polymerase (RdRp), which lacks the proofreading activity (13, 14). The long-term arthropod-virus coevolution shaped the complicated interactions between them, leading to the development of highly specialized transmission mechanisms in a wide range of hosts, increasing virus transmission patterns (15). Moreover, mosquitoes are infected by a wide variety of viruses with a restricted host range, referred to as insect specific viruses (ISVs), which do not infect vertebrates but are associated with persistent and asymptomatic infections in their reservoirs. ISVs have attracted considerable attention in recent years due to their potential role in modulating mosquito biology and influencing transmission of pathogenic arboviruses (16, 17). ISVs, like many other arboviruses, belong to the RNA virus families and constitute a key component of the mosquito virome. Their widespread presence across different arthropod species and geographic regions highlights their ubiquity and potential influence on arbovirus ecology and transmission dynamics (16). Indeed, ISVs have been implicated in shaping the ecological interactions between mosquitoes and arboviruses, as reported by some studies suggesting that ISVs may interfere with arboviral replication, potentially reducing the ability of mosquitoes to transmit pathogenic viruses, such as WNV (18). Furthermore, some ISVs appear to be vertically transmitted without apparent negative fitness consequences on arthropod vectors, effectively becoming endogenous in their arthropod hosts (19). Understanding the epidemiology, transmission dynamics, and pathogenesis of mosquito-borne viruses is crucial for developing effective surveillance, prevention, and control strategies. The rapid development of next-generation sequencing (NGS) technology enabled the non-specific detection of viruses in mosquitoes and promoted the surveillance for viromes (20).

In the present study, we analyzed the virome of *Culex pipiens, Aedes albopictus,* and species of Psychodidae Phlebotominae from the Siena and Grosseto districts, in Central-Southern Tuscany, Italy, using a metagenomic approach.

## MATERIALS AND METHODS

### Mosquito sampling and identification

A total of 3,732 mosquitoes and sandflies were field-collected with BG-Pro CDC light traps (Biogents AG, Regensburg, Germany) equipped with insect-attracting UV LED lights and baited with dry ice to simulate the CO₂ produced by host respiration, which were activated overnight, from sunset to sunrise. These traps were placed at various urban and peri-urban sites in the Siena and Grosseto districts (Tuscany, Italy) during the spring-early autumn season (May–October) of the 2022–2024 period. The collected specimens were kept alive within a net inside the trap and immediately transported to the laboratory for sorting and identification. In parallel, oviposition traps were also used as a sampling method. Water-filled canisters were placed in similar environmental settings described above and monitored daily for the presence of mosquito eggs and larvae. Once larvae were detected, the containers were transferred to the laboratory and maintained at room temperature to monitor larval development. To accelerate development, protein-based animal food was added to the water. Under these conditions, larval development lasted approximately 15 ± 5 days. The rearing containers were inspected daily, and emerged adults were collected using an entomological aspirator. The collection of adult *A. albopictus* mosquitoes was instead conducted on site with a net. Adult mosquitoes were sorted and identified through morphological examination using a Zeiss Axio Zoom V16 stereomicroscope. Specimen identification was primarily based on Snow et al. and Severini et al. at the Laboratory of Evolutionary Zoology and Systematics of the University of Siena (Siena, Italy) (21, 22). When possible, female or male/female mosquitoes were pooled in groups of 1–50 individuals based on date of collection. Pools were frozen at −80°C until used. Then, mosquitoes were thawed at room temperature and diluted in Dulbecco’s Modified Eagle Medium (EuroClone, Milan, Italy) supplemented with antibiotics (penicillin and streptomycin; EuroClone) to a final concentration of 1 individual/20 µL. Each pool was then mechanically homogenized for five min using a grinder pestle (Merck Millipore, Milan, Italy) to release virus particles from mosquito corps. The resulting mosquito homogenates were centrifuged at 14,000 × *g* for 10 min at 4°C, then cleared supernatants were aliquoted and stored at −80°C or immediately processed for nucleic acid extraction and virus isolation.

### Nucleic acid extraction and reverse transcription

Total RNA was extracted from a 50 µL aliquot of each mosquito or sandfly pool homogenate by using the RNeasy Plus Mini Kit (Qiagen, Milan, Italy), according to the manufacturer’s instructions. Removal of host genomic DNA was achieved with unique eliminator columns (provided with the kit), while most RNAs <200 nucleotides (such as rRNAs and tRNAs) were excluded due to silica cut-off, leading to a highly pure extract consisting mostly of messenger RNA or viral RNA. Resulting total RNAs were recovered in 50 µL of RNase-free water and tested for phleboviruses and flaviviruses. Twenty-five microliters of reaction volume were set up by using the 1-Step Go reverse-transcription polymerase chain reaction (RT-PCR) Kit (PCRBiosystems, London, UK) with the pan-Phlebovirus degenerated PhlP2/PhlM2 primers targeting the RdRp gene (23), the PhlN_Fw/PhlN_Rv primers targeting the Phlebovirus nucleoprotein (N) gene (24), and the pan-Flavivirus degenerated PF1S/PF2R-bis primers targeting the RNA-dependent RNA polymerase (NS5) gene (25). Ten microliters of RT-PCRs were analyzed by agarose gel electrophoresis. The expected amplicon size was 507 base pairs (bp) (RdRp), 309 bp (N), and 197 bp (NS5), respectively.

### Sample sequencing

Samples that tested positive for one or both viral targets of phleboviruses and/or flaviviruses, by RT-PCR, were selected for metagenomic analysis. Total RNA isolated from arthropod homogenates, as previously described, was quantified with the Qubit RNA high sensitivity assay kit and the Qubit 3.0 fluorometer (Thermo Fisher Scientific, Rodano, Italy). The cDNA synthesis and the library preparation were generated using the NEBNext Single Cell/Low Input RNA Library Prep kit for Illumina (New England Biolabs, Milan, Italy) according to the manufacturer’s protocol. Whether the RNA samples were quantifiable, 100 ng total RNA were used in the reverse-transcription (RT) reaction for cDNA synthesis; otherwise, the maximum volume was used. The obtained library was then quantified with the Qubit DNA high-sensitivity assay kit and the Qubit 3.0 fluorometer (Thermo Fisher Scientific). The median size of the library fragments was assessed with the 4150 TapeStation instrument (Agilent Technologies, Milan, Italy) with a D1000 kit (Agilent Technologies) following the manufacturer’s recommendations. Normalized libraries were diluted to a 10 pM equimolar concentration, pooled, and denatured. Paired-end sequencing was performed using the MiSeq platform (Illumina, Milan, Italy) using a MiSeq Reagent Micro Kit v2 (300-cycles) or MiSeq Reagent Nano Kit v2 (500-cycles), based on the number of samples processed (Illumina, Milan, Italy).

### Sequence assembly and analysis

FASTQ files were analyzed using the open source CzID platform (https://czid.org) (26). The FASTQ files were uploaded on the platform, and the Illumina metagenomic pipeline was launched, selecting the Bowtie2 algorithm and “Mosquitoes” as the reference host. Then, Double Index Alignment of Next Generation Sequence Data set (DIAMOND) was used for the alignment against the NCBI’s non-redundant database (27). Specifically, reads were first filtered for low complexity using the LZW filter, low-quality bases (Phred < 17), and for short read length (<35 bp). Viral sequences were then retrieved considering the indicated filter parameters: e-value ≤1 × 10^−5^, minimum alignment length ≥50 amino acids, minimum identity ≥30%. In the reporting stage, we considered only taxa supported by both NT and NR alignments (nucleotide + protein concordance) and with alignment length >50 bp and reads per million >1, which effectively reduces spurious hits and overclassification. The annotated FASTA was then generated and further assembled with SPADES software. The resulting reads were then aligned with BLAST, and the new annotated FASTA was generated. The unused reads were collected in an unidentified folder and could be used for manual analysis.

### Phylogenetic analysis

The consensus sequences more frequently identified and with a coverage no lower than 30% were selected and uploaded to the Geneious software (version 2025.0.2), and the alignment was performed with the MUSCLE algorithm, using the default parameters (three iterations and gap opening penalties). The pairwise distances were automatically calculated by the software. The phylogenetic tree was generated using the neighbor-joining algorithm using the Jukes–Cantor distance model, which assumes equal and constant mutation rates between all nucleotide pairs. Bootstrapping and reconstitution were carried out with 1,000 replicates to obtain the confidence level of the phylogenetic tree. The cutoff was set to 50 while other parameters were as default. The GenBank accession numbers relative to reference sequences used for phylogenetic analysis are the following: Culex pipiens-associated Tunisia virus OQ968278.1, MW434967.1, NC_040723.1, MG457155.1, MG457154.1; Wenzhou Sobemo-like virus 4 MT096519.1, MT591567.1, ON918610.1; Culex iflavi-like virus 4 NC_040832.1, NC_040716.1, NC_040574.1; *A. albopictus* anphevirus MW147277.1, LC778999.1, OR715784.1, OR729834.1; Picorna-like virus MG833031.1, NC033238.1, NC032990.1, NC033224.1, NC032772.1, NC032778.1, NC033214.1, KX883896.1; and *Aedes* Phasmavirus MT361043.1.

## RESULTS

**Fig 1:**
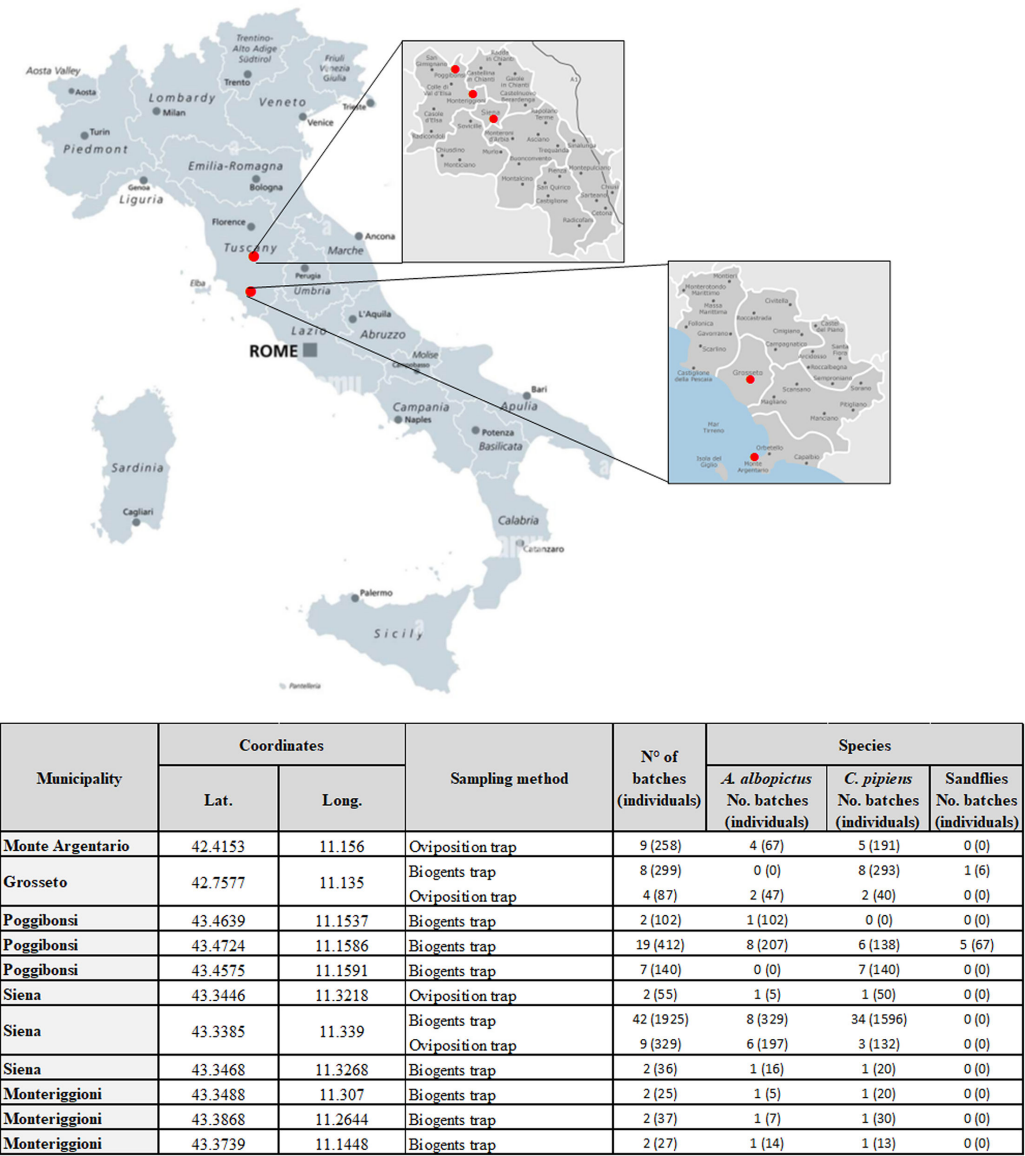
Geographical overview of sampling sites and applied collecting methods. Geographical map showing sampling locations in Central-Southern Tuscany, Italy, covering the Siena (Siena, Monteriggioni, and Poggibonsi) and the Grosseto municipalities (Grosseto and the Argentario Peninsula). Embedded table provides geographical coordinates of each collecting site, specific collecting methods, and the number of pools/individuals collected from each site.

Mosquito and sandfly samples were collected during the spring-summer-autumn period (May–October) of the 2022–2024 years lapse. Sampling was conducted at various sites located in peri-urban or rural areas of the Siena and Grosseto provinces (Fig. 1). In total, 3,732 individuals were collected in the indicated areas and species (21, 22). Among them, 26.7% (*n* = 996/3,732) were identified as *A. albopictu*s, 71.4% (*n* = 2,623/3,732) as *C. pipiens,* and 2% (*n* = 73/3,732) as representatives of the sandfly subfamily Phlebotominae (Table 1). Annual distribution of mosquito and sandfly evidenced an environmental circulation peak for the *A. albopictu*s during the 2023 season (*n* = 579/1,399, 41.2%), for *C. pipiens* during the 2022 season (*n* = 1,734/2,082, 83.3%), and sandflies (Phlebotominae gen. sp.) during the 2024 season (*n* = 29/251, 11.6%) (Table 1). On the basis of arthropod species, sex, and capture areas, collected individuals were divided into a total of 110 pools based on taxonomic classification (mosquito or sandfly species), sex, and capture areas (Table 1; Table S1). To investigate the presence of phleboviruses and flaviviruses vehicle by the indicated samples, an RT-PCR screening was set up based on the use of degenerated primers, targeting the RNA-dependent RNA-polymerase (L gene) of phleboviruses and flaviviruses (NS5 gene). At the same time, since possible reassortments between phleboviruses might occur, an RT-PCR was also performed to detect the presence of the Small (S) phlebovirus segment by the amplification of a portion of the nucleoprotein (N) gene. The RT-PCR analyses performed on RNA purified by the arthropod pools yielded qualitative positive results for numerous batches (*n* = 67/110) during the 2022–2024 period. Considering the positivity rate for phleboviruses and/or flaviviruses, the highest rate was observed in the 2024 season, with an incidence of 12.5% (*n* = 2/16) for both phlebovirus RdRp and N targets and 56.3% (*n* = 9/16) for flavivirus NS5 gene (Table 2). In the same period, a high number of arthropod batches positive for only one of the phlebovirus targets (RdRp 37.5%, *n* = 6/16 or N 25%, *n* = 4/16) was also reported, likely associated with the presence of recombinant viruses or phleboviruses that share sequence homology with only one of the specific primer targets. Additionally, in 18.8% (*n* = 3/16) of cases, both phlebovirus and flavivirus genomes were observed, suggesting co-infection of the arthropod vectors (Table 2). The highest rate of positive samples was observed in 2024, despite the lowest number of individuals collected (*n* = 251, Table 2). Indeed, the incidence of phleboviruses, positive for both viral targets, was 12.5% (*n* = 2/16), for only one target was 37.5% for RdRp (*n* = 6/16) and 25% for N (*n* = 4/16), while that of flaviviruses was 56.2% (*n* = 9/16) (Table 2). Additionally, the highest number of flaviviruses and phleboviruses (positive for at least one genome target) co-infected specimens was recorded during the same year (*n* = 3/16, 18.8%) (Table 2). The 2024 season showed the highest detection rates of phleboviruses in *A. albopictus* (16.7%, *n* = 1/6), compared to other vector species (Table 2). In contrast, *C. pipiens* specimens showed the highest positivity for flaviviruses (*n* = 5/7, 71.4%). Moreover, several Phlebotominae spp. pools were positive for RdRp or N targets, with higher detection rates than in previous seasons (RdRp: *n* = 2/3, 66.7%; N: *n* = 1/3, 33.3%; both RdRp and N: *n* = 1/3, 33.3%) (Table 2). In this context, it is important to underline that in the 2022 season, Phlebotominae spp. captures were negligible (Table 1).

**TABLE 1:** Samples summary and identification^*a*^

	2022		2023		2024		Total	
	No. of batch	No. of specimens (%)	No. of batch	No. of specimens (%)	No. of batch	No. of specimens (%)	No. of batch	No. of specimens (%)
*A. albopictus*	9	328 (15.8)	19	576 (41.2)	6	92 (36.7)	34	996 (26.7)
*C. pipiens*	40	1,734 (83.3)	23	799 (57.1)	7	130 (51.8)	70	2,663 (71.4)
Phlebotominae spp.	1	20 (1)	2	24 (1.7)	3	29 (11.6)	6	73 (2)
Total	50	2,082	44	1,399	16	251	110	3,732

^*a*^ Based on identification, sex, and collecting area, individuals were pooled each year to a maximum of 50 individuals per batch. Alongside, the circulation of each insect species was calculated with respect to the number of individuals collected during the corresponding season.

**TABLE 2:** Results of the RT-PCR screening on arthropod samples^*a*^

	2022	2023	2024
Viral target	No. of positive pools (%)	No. of positive pools (%)	No. of positive pools (%)
Phlebovirus RdRp	13 (26)	7 (15.9)	6 (37.5)
Phlebovirus N	4 (8)	3 (6.8)	4 (25)
Flavivirus NS5	6 (12)	5 (11.4)	9 (56.3)
Phlebovirus RdRp and N	1 (2)	0 (0)	2 (12.5)
Phlebovirus and Flavivirus	1 (2)	3 (6.8)	3 (18.8)

	2022				
	Phlebovirus RdRp No. of positive (%)	Phlebovirus N No. of positive (%)	Flavivirus No. of positive (%)	Phlebovirus RdRp and N No. of positive (%)	Phlebovirus and Flavivirus No. of positive (%)
*A. albopictus*	1/9 (11.1)	1/9 (11.1)	3/9 (33.3)	0/9 (0)	0/90 (0)
*C. pipiens*	12/40 (30)	3/40 (7.5)	3/40 (7.5)	1/40 (2.5)	1/40 (2.5)
Phlebotominae spp.	0/1 (0)	0/1 (0)	0/1 (0)	0/1 (0)	0/1 (0)
Total	13/50 (26)	4/50 (8)	6/50 (12)	1/50 (2)	1/50 (2)

	2023				
	Phlebovirus RdRp No. of positive (%)	Phlebovirus N No. of positive (%)	Flavivirus No. of positive (%)	Phlebovirus RdRp and N No. of positive (%)	Phlebovirus and Flavivirus No. of positive (%)
*A. albopictus*	3/19 (15.8)	2/19 (10.5)	5/19 (26.3)	0/19 (0)	3/19 (15.8)
*C. pipiens*	3/23 (13)	1/23 (4.3)	0/23 (0)	0/23 (0)	0/23 (0)
Phlebotominae spp.	1/2 (50)	0/2 (0)	0/2 (0)	0/2 (0)	0/2 (0)
Total	7/44 (15.9)	3/44 (6.8)	5/44 (11.4)	0/44 (0)	3/44 (6.8)

	2024				
	Phlebovirus RdRp No. of positive (%)	Phlebovirus N No. of positive (%)	Flavivirus No. of positive (%)	Phlebovirus RdRp and N No. of positive (%)	Phlebovirus and Flavivirus No. of positive (%)
*A. albopictus*	2/6 (33.3)	3/6 (50)	3/6 (50)	1/6 (16.7)	1/6 (16.7)
*C. pipiens*	2/7 (28.6)	0/7 (0)	5/7 (71.4)	0/7 (0)	1/7 (14.3)
Phlebotominae spp.	2/3 (66.7)	1/3 (33.3)	1/3 (33.3)	1/3 (33.3)	1/3 (33.3)
Total	6/16 (37.5)	4/16 (25)	9/16 (56.2)	2/16 (12.5)	3/16 (18.8)

^*a*^ The results obtained from the RT-PCR screening of the insect batches are presented as target-specific incidence, expressed as the number (No.) and percentage of positive pools out of the total pools, during each specific year, and the No. and percentage of positive pools categorized by species incidence for each collection period.

### Virome of *A. albopictus*, *C. pipiens,* and sandflies collected in Tuscany, Italy

From the total of 67 pools positive by RT-PCR for phleboviruses and/or flaviviruses, 42 samples were selected for metagenomic sequencing analysis. Among them, only 35 pools generated viral reads; 19 pools (54.3%) were derived from *C. pipiens*, 12 (34.3%) from *A. albopictus,* and 4 (11.4%) from Phlebotominae spp. Furthermore, six pools were composed of *A. albopictus* or *C. pipiens* reared in the lab from larvae collected in Siena and Grosseto districts. High-throughput sequencing on the MiSeq (Illumina) platform generated more than 44 million reads across all pools. The FASTQ files, analyzed by the open-source Czid platform and the Illumina metagenomic pipeline, showed significant similarity to viral-origin sequences in 28.6% (12.6 million) of the total reads. Viruses from 14 different families were detected in these samples, with a notable portion of them remaining unclassified (Table 3). The most frequently detected virus genera belonged to *Iflaviridae*, *Phasmaviridae, Tombusviridae, Xinmoviridae, Mesoniviridae, Phenuiviridae,* and *Nodaviridae* families. However, a high number of analyzed sequences were recorded as unclassified virus order/family in GenBank Taxa (Table 3). Full report of sequenced samples, including NGS reports and sample details, is provided in Table S1. Further characterization of unidentified reads (30 million) in the samples is required to determine whether they contain novel unknown viral genomes, although reads mapping with a low identity to the virus database were already analyzed.

**TABLE 3:** Summary of one-shot sequencing results^*a*^

Sample	Virus	Virus family/order	Sample	Virus	Virus family/order
GRC9	Culex iflavi-like virus	iflaviridae	Pool 12	Culex iflavi-like virus 4	iflaviridae
	Culex pipens-associated Tunisia virus	Unclassified Riboviria		Hubei picorna-like virus 63	Unclassified Riboviria
	Hubei mosquito virus	Unclassified Riboviria		Yongsan iflavirus 1	iflaviridae
	Rinkaby virus	Negevirus	Pool 15	Hubei picorna-like virus 41	Unclassified Riboviria
GRC8	Wallerfield virus	Negevirus		Ortophasmavirus barstukasense	Phasmaviridae
	Daeseongdong virus 2	Unclassified		Wenzhou sobemo-like virus 4	Unclassified Riboviria
GRC6	Culex picorna-like virus	Picornavirales	Pool 17	Culex pipens-associated Tunisia virus	Unclassified Riboviria
	Culex pipens-associated Tunisia virus	Unclassified Riboviria	Pool 19	Phlebovirus Puniqueense	Phenuiviridae
GRC5	Culex iflavi-like virus	iflaviridae	Pool 22	Wenzhou sobemo-like virus 4	Unclassified Riboviria
	Culex picorna-like virus	Picornavirales		Aedes albopictus anphevirus	Xinmoviridae
	Picornaviridae	Picornaviridae	Pool 25	Aedes-ifla-like virus	iflaviridae
	Culex pipens-associated Tunisia virus	Unclassified Riboviria	Pool 26	Wenzhou sobemo-like virus 4	Unclassified Riboviria
SM3	Aedes phasmavirus	Phasmaviridae	Pool 20	Culex-associated Tombus-like virus	Tombusviridae
	Guangzhou-sobemo-like virus	Solemoviridae		Culex pipens-associated Tunisia virus	Unclassified Riboviria
	Wenzhou sobemo-like virus 4	Unclassified Riboviria		Culex mosquito virus 1	Nodaviridae
ARA3	Aedes phasmavirus	Phasmaviridae		Wuhan mosquito virus 4	Orthomyxoviridae
GRC3	Culex pipens-associated Tunisia virus	Unclassified Riboviria	Pool 27	Aedes albopictus anphevirus	Xinmoviridae
ARA4	Vulex iflavi-like virus	iflaviridae		Wenzhou sobemo-like virus 4	Unclassified Riboviria
PHL1	Culex-associated Tombus-like virus	Tombusviridae	Pool 24	Aedes ifla-like virus	iflaviridae
PGACC2	Aedes albopictus anphevirus	Xinmoviridae		Sonnbo virus	Partitiviridae
FFC5F	Culex pipens-associated Tunisia virus	Unclassified Riboviria	Pool 28	Culex pipens-associated Tunisia virus	Unclassified Riboviria
SM2	Aedes ifla-like virus	iflaviridae		Culex iflavi-like virus 4	iflaviridae
	Daeseongdong virus 2	Unclassified		Culex daeseongdong-like virus	Nodaviridae
	Espirito santo virus	Birnaviridae	Pool 29	Culex daeseongdong-like virus	Nodaviridae
PGACC	Aedes albopictus anphevirus	Xinmoviridae	CO1	Aedes albopictus anphevirus	Xinmoviridae
	Aedes phasmavirus	Phasmaviridae		Aedes phasmavirus	Phasmaviridae
	Aedes flavivirus	Flaviviridae		Wenzhou sobemo-like virus 4	Unclassified Riboviria
MC6	Aedes ifla-like virus	iflaviridae	PG6	Culex pipens-associated Tunisia virus	Unclassified Riboviria
	Aedes albopictus anphevirus	Xinmoviridae	PG8	Hubei diptera virus 17	Unclassified Riboviria
	Wenzhou sobemo-like virus 4	Unclassified Riboviria	SM4	Culex pipens-associated Tunisia virus	Unclassified Riboviria
FFA1M	Aedes albopictus anphevirus	Xinmoviridae	MC1	Wallerfield virus	Negevirus
	Aedes phasmavirus	Phasmaviridae	MC3	Wallerfield virus	Negevirus
	Aedes flavivirus	Flaviviridae	SL1	Wallerfield virus	Negevirus
	Wenzhou sobemo-like virus 4	Unclassified Riboviria	BDA	Aedes albopictus anphevirus	Xinmoviridae

^*a*^ List of viruses and relative taxa identified by metagenomic analysis of insect pools.

**Fig 2:**
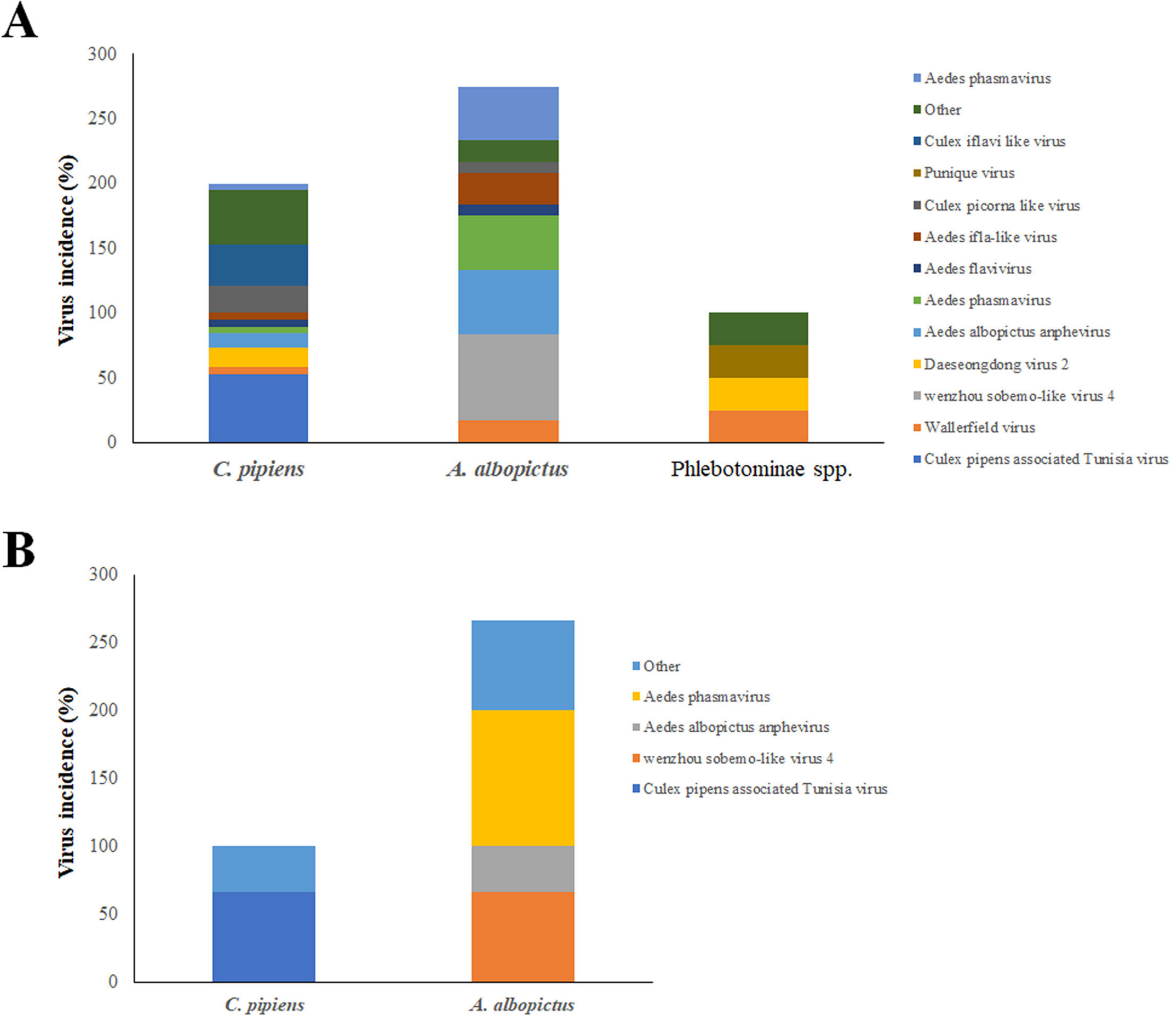
Contribution of virus genus to the virome of the three different arthropod species. The virome of arthropods collected in Tuscany was inferred by metagenomic analysis, reporting the incidence (%) of the most frequently identified viruses, in (**A**) adults or (**B**) in-laboratory reared species.

**Fig 3:**
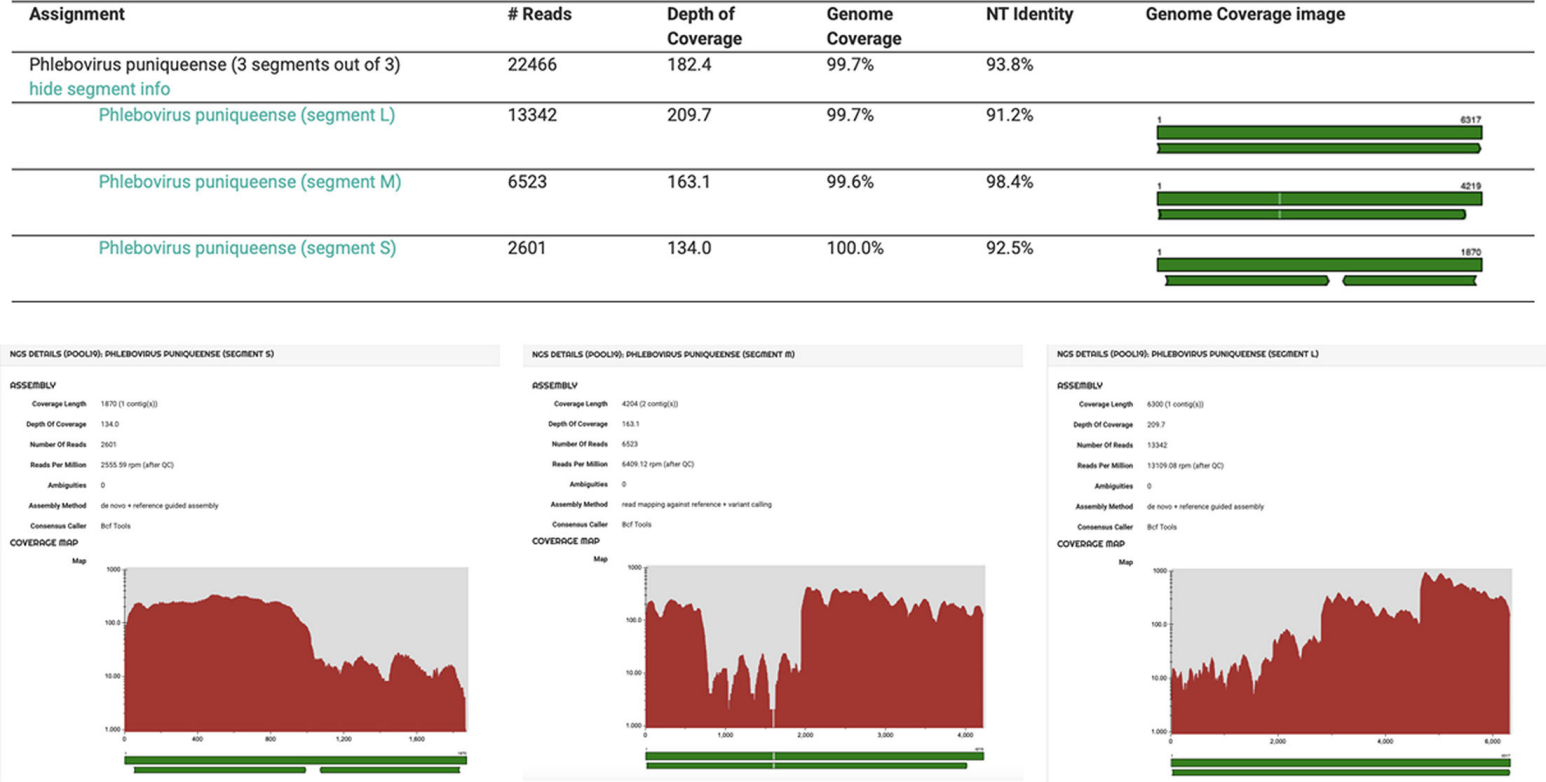
Punique virus coverage profile. Analysis of consensus sequences of the three genomic segments S, M, and L from Punique virus obtained by NGS analysis of Phlebotomine spp. Pool 19. Sequences’ quality scores are reported together with the reference strain present in the GenBank.

We then performed a comparative analysis of the virus species contributions to the virome of the arthropod species herein investigated to assess species-specific viral diversity. Our results demonstrated that both mosquito species harbor a variety of virus genera belonging to different viral families, with notable differences in the composition and frequency of them. Viral families more frequently identified included the *Iflaviridae/Flaviviridae* (*n* = 12/35; 34.3%), being detected in both *C. pipiens* and *A. albopictus* samples, with a frequency of 66.7% (*n* = 8/12) and 33.3% (*n* = 4/12), respectively. Further analysis of consensus, generated by metagenomic sequencing, revealed a high incidence (*n* = 8/35; 22.9%) for the Wenzhou sobemo-like virus 4, which appears to be exclusively transmitted by the *A. albopictus* species (*n* = 8/8; 66.7%) (Fig. 2A). Moreover, this virus was detected in male individuals reared in the lab from larvae (*n* = 2/6; 33.3%), indicating a possible transovarial transmission (Fig. 2B). Similarly, Culex pipiens-associated Tunisia virus was identified in a significant proportion (*n* = 10/35; 28.6%) of batches subjected to NGS. According to the taxonomic name of the virus, it was exclusively found in the *C. pipiens* genus (*n* = 10/19; 52.6%) and its transovarial transmission was hypothesized, being detected in 2/3 batches (66.7%) of in-lab reared samples (Fig. 2A and B). Other viruses identified with a high prevalence included Aedes Phasmavirus (*n* = 6/35, 17.1%), Aedes Anphevirus (*n* = 8/35, 22.9%), Wallerfield virus (*n* = 4/35, 11.4%), Daeseongdong virus (*n* = 4/35, 11.4%), and viruses belonging to the Picornavirales order (*Iflaviviridae* family) (*n* = 11/35, 31.4%) (Fig. 2A). Overall, the host reservoirs for these viruses were quite variable. Specifically, some ISVs were predominantly detected in *A. albopictus*, such as Phasmavirus, Anphevirus, and Wallerfield virus, while others were found preferentially in *C. pipiens* (Daeseongdong virus and *Picornaviridae*). Only a minority of viruses were endogenous to Phlebotominae spp. (*n* = 2/35, 5.7%) (Fig. 2A). Moreover, the detection of Phasmavirus and Anphevirus in individuals reared from larvae further supported a possible mechanism for their persistence in the ecosystem through transovarial transmission (Fig. 2B). It is worth noting that the Punique virus (PUNV), belonging to the Sandfly Fever Naples serogroup viruses, widely distributed in North Africa (Tunisia and Algeria) and capable of infecting humans, was identified in a single Phlebotominae spp. pool (*n* = 1/4; 25%) (Fig. 2A) (28–30). Metagenomic analysis revealed the presence of the virus with a mean coverage of 99.6% ± 0.21% and a mean depth of 168.9X ± 38.2, including the three genomic segments (S, M, and L) (Fig. 3).

Blast analysis of the Italian PUNV genome sequence (GenBank Accession Nos. PQ569738.1, PQ569737.1, and PQ569736.1) showed a great homology with the PI-B4-2008 strain isolated in Tunisia in 2008. These data demonstrate the presence and circulation of the Punique virus in Italy for the first time.

**Fig 4:**
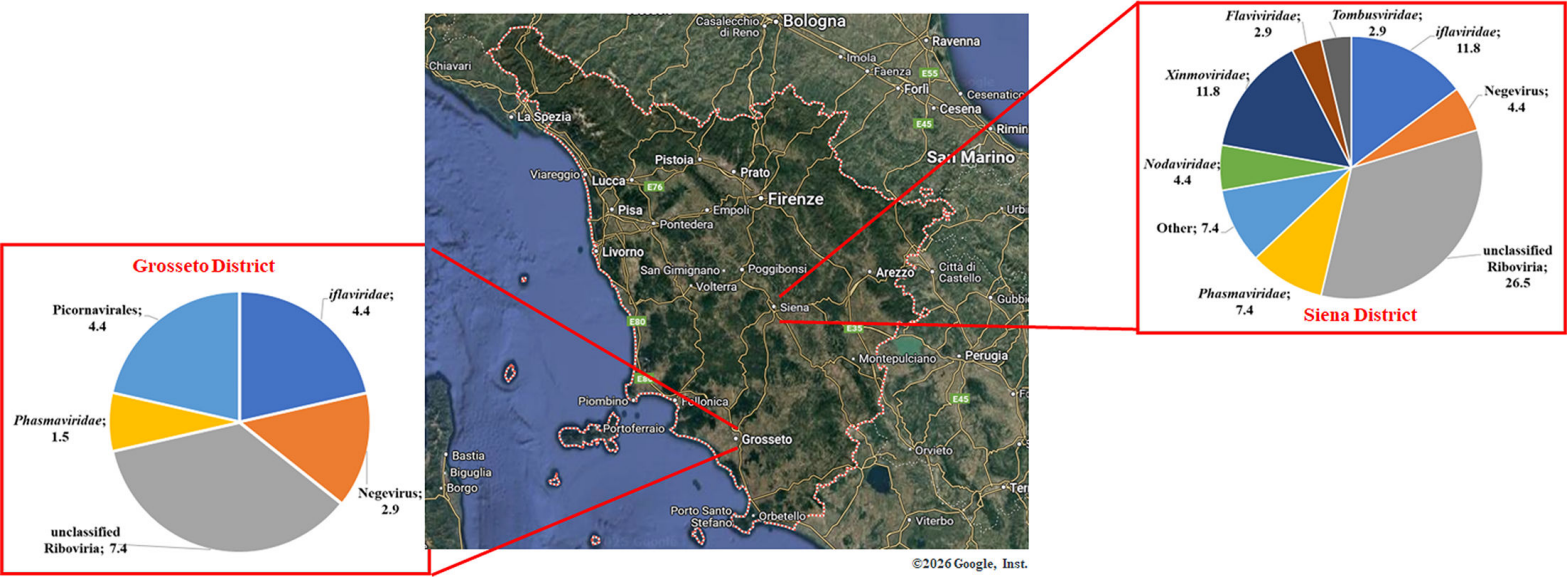
Geographical comparison of the mosquito virome. Based on NGS-derived sequences from mosquito samples collected in the Siena and Grosseto districts, the presence of each virus family was estimated and expressed as a percentage and presented as a pie chart.

The geographical distribution of the main virus families identified by NGS revealed differences in the mosquito virome between the two Tuscany districts investigated (Siena and Grosseto). Specifically, a more diverse range of ISVs was detected in samples from the Siena district compared to those from Grosseto. In detail, members of the *Flaviviridae*, *Nodaviridae*, *Tombusviridae*, and *Xinmoviridae* families appeared to colonize *A. albopictus* and *C. pipiens* in the Siena district, as they were not detected in samples from Grosseto. Conversely, Picornavirales-associated contigs were found exclusively in samples from Grosseto. Nevertheless, *Iflaviridae*, Negevirus, Riboviria, and *Phasmaviridae* were present in both areas, although their impact on the mosquito virome composition varied (Fig. 4). Despite virome analysis performed on sequenced samples, the presence of non-viral reads, which were mainly classified as bacteria domain, was also detected. The composition of the bacterial community was various and differed in each mosquito host species. Although bacterial analysis was not the aim of this study, it was interesting to confirm the presence of *Wolbachia* spp. (Alphaproteobacteria), a genus of common obligate intracellular parasites of invertebrate taxa (31), in most samples (*n* = 18/35; 51.4%), as indicated by 16s rRNA contigs recovered. Although this bacterium was identified with higher prevalence in *C. pipiens* pools (*n* = 12/19; 63.1%), it was also detected in *A. albopictus* batches (*n* = 6/12; 50%) in this analysis. By contrast, no reads were found in sandfly pools.

### Phylogenetic analysis

**Fig 5:**
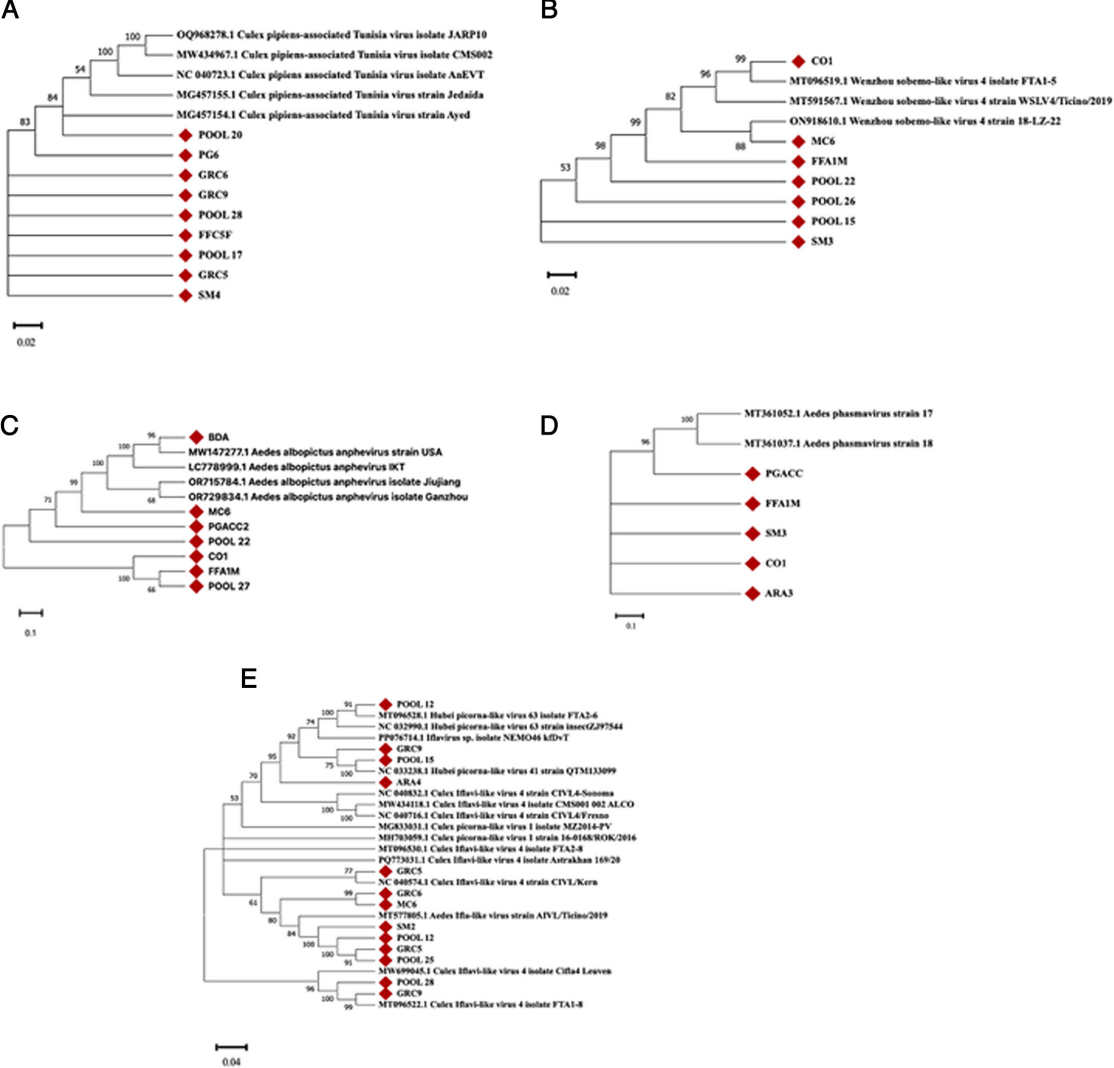
Phylogenetic analysis of virus whole-genome sequences identified by NGS sequencing. Phylogenetic tree of (**A**) Culex pipiens-associated Tunisia virus, (**B**) Wenzhou Sobemo-like virus 4, (**C**) *Aedes albopictus* anphevirus, (**D**) Aedes Phasmavirus, and (**E**) Picornavirales. Values at nodes represent the bootstrap values. The scale bar indicates the number of nucleotide substitutions per site. Names of sequenced samples are mentioned, along with the GenBank accession number for each reference, and represented by a solid red diamond.

Phylogenetic analysis was performed on whole-genome sequences for those viruses with a genome coverage higher than 70% and more represented in the investigated samples. Among them, Culex pipiens-associated Tunisia Virus, Whenzou sobemo-like virus 1, sequences mapping to *Picornavirales* order (Culex/Aedes iflavi-like virus and picorna-like viruses), Aedes albopictus anphevirus, Aedes Phasmavirus, and Culex picorna-like virus were selected and further analyzed. Using the Geneious Prime software (version 2025.0.2), consensus sequences were aligned using the MUSCLE algorithm with a minimum GC content of 50% and following the default parameters proposed by the software. The phylogenetic distances between the recovered virus consensus sequences and the relative references available on the GenBank database were calculated and shown in Table S2. Phylogenetic trees were then constructed based on the neighbor-joining method for the previously mentioned viruses. The phylogenetic analysis results are presented in Fig. 5. In detail, we identified nine sequences mapping to the Culex pipiens-associated Tunisia virus genome. The phylogenetic tree of these samples showed that most of the Italian sequences clustered with viral strains isolated in Tunisia (GenBank Accession Nos. MG457155.1 and MG457154.1) (Fig. 5A). By contrast, none of the Italian samples clustered under the other reference sequences with different geographical origins, such as Brazil, the U.S.A., and Vietnam (GenBank Accession Nos. OQ968278.1, MW434967.1, and NC040723.1) (Fig. 5A). Regarding the Wenzhou sobemo-like virus 4, the majority of samples (*n* = 5/7, 71.4%) formed a separate cluster distinct from the deposited reference sequences (Fig. 5B). Two remaining samples (CO1 and MC6), instead, showed a close homology with strains isolated in Spain and China (GeneBank Accession Nos. MT096519.1 and ON918610.1, respectively) (Fig. 5B). The evolutionary relationships among the identified Aedes albopictus anphevirus were further investigated. The phylogenetic tree showed two major clusters: one consisting of the U.S.A. and Japan strains (GeneBank Accession Nos. MW147277.1 and LC778999.1, respectively) and the other one grouping Chinese isolates (GeneBank Accession Nos. OR715784.1 and OR729834.1). Six out of seven Italian samples formed distinct clusters with moderate to high bootstrap values (66–100), which, however, were more closely evolutionary related to the Chinese strains (Fig. 5C). The only exception was represented by the BDA sample, which clustered under the U.S.A. strain (d = 0.002) (Fig. 5C). Ultimately, five sequences were assigned to the Aedes Phasmavirus genome, the majority of which (*n* = 4/5; 80%) formed a separate cluster different from the Chinese references (GenBank Accession Nos. MT361043.1 and MT361052.1). By contrast, only the PGACC sample showed high similarity with the two reference sequences that are closely related to each other (Fig. 5D). Sequences retrieved from several samples, aligned to the Picornavirales order, including Picorna-like viruses, Culex/Aedes Iflavi-like viruses, or both. Regarding the Iflavi-like viruses, the phylogenesis revealed high similarity among most of the Italian samples (*n* = 6/10; 60%) to the Iflavi-like virus Swiss strain (GeneBank Accession Nos. MT577805.1) with high bootstrap support (≥80) and average distance d = 0.01 (Fig. 5E). By contrast, two samples were more similar to the FTA1-8 Spain lineage (d = 0.014 and d = 0.007) (GeneBank Accession Nos. MT096522.1) and to the Belgian strain (GeneBank Accession Nos. MW699045.1) (d = 0.016 and d = 0.009) (Fig. 5E). On the other hand, only one sample (GRC5) clustered under the strain Kern isolated in the U.S.A. (d = 0.014) (GenBank Accession Nos. NC_040574.1). Moreover, sequence alignment mapping to the Picorna-like virus genome showed that all samples (GRC9, pool 15, ARA4, and pool 12) were classified as different Hubei picorna-like viruses isolated in different countries, such as China and Spain (GenBank Accession Nos. NC033238.1 and MT096528.1, respectively) (Fig. 5E). Regarding the Daeseongdong virus 2, although its high incidence among sequenced samples (*n* = 4/35; 11.4%), a sequence coverage lower than 70% was obtained for most samples, phylogenetic analysis was not performed. However, BLAST alignment results of selected sequences showed that all samples shared high sequence identity (98.4%–99%) with Daeseongdong-like virus strain A12.2549/ROK/2012 isolated in South Korea (GenBank Accession No. NC028489.1).

## DISCUSSION

Mosquitoes are the principal vectors of arboviruses, posing significant threats to human health. To investigate the diversity of viruses associated with these vectors, we performed high-throughput RNA sequencing on mosquito and sandfly populations collected over three years (2022–2024) in the Siena and Grosseto districts of Central-Southern Tuscany, Italy. The study focused on three key species: *A. albopictus*, *C. pipiens*, and Phlebotominae spp., selected for their abundance and vector potential in the region (32–36). A total of 3,732 adult arthropods, field-collected or laboratory-reared from larvae, were sampled. Based on taxonomy, sex, and collection area, the samples were pooled and screened by RT-PCR using pan-degenerate primers targeting flaviviruses and phleboviruses (23–25). Sixty-seven pools tested positive, of which 42 were selected for metagenomic sequencing. Multiple ISVs from diverse viral taxa were identified in 35 processed arthropod pools. The remaining seven pools tested negative, supporting the reliability of the analysis and excluding potential cross-contamination or misinterpretation of results. Some viruses were found across mosquito genera, while others were genus-specific. Furthermore, some virus species were found to be characteristic of the mosquito virome from two different collecting sites, about 80 km apart (Siena and Grosseto districts). On the other hand, viruses within several virus families were detected in samples from both collecting areas, with a limited genetic divergence, suggesting that the viruses were shared among mosquito populations circulating in the Central-Southern Tuscany region. Our findings revealed that both mosquito species have different virus genera with significant differences in their composition and frequency. Among the ISVs, Aedes phasmavirus, Aedes anphevirus, Culex iflavi-like virus 4, and viruses from the *Picornaviridae* family were more prevalent. These viruses were detected in both *C. pipiens* and *A. albopictus*, suggesting possible intergeneric transmission. Regarding the Italian Aedes anphevirus, samples were clustered in a distinct clade closely related to Asian rather than American strains (Fig. 4C). A recent study showed that Aedes Anphevirus may reduce DENV replication in cell culture (37). Very few data are available about the *Phasmaviridae* family virus ecology and worldwide distribution (38, 39). Interestingly, in our study, the Phasmavirus genome was detected in both larvae and adults of *A. albopictus*, suggesting a potential transovarial transmission route. However, since ovitraps collect water and organic material from natural breeding habitats, they may also accumulate environmental contaminants, including viral particles present in the surroundings. Therefore, detection of viral RNA or particles in ovitrap-collected samples does not necessarily indicate true infection or vertical transmission within mosquitoes (40, 41). Despite limited data about *Phasmaviridae* in mosquitoes, members of this viral family have been previously identified in Sweden and Greece. Our identification of *Phasmaviridae* viruses in adult species of both *Aedes* and *Culex* spp. provides new insight into their geographical distribution (42, 43). Furthermore, phylogenetic analysis revealed that Italian Phasmavirus samples formed distinct clusters from the Chinese reference strains, suggesting the ongoing virus genetic evolution. Moreover, we identified picorna-like viruses within the *Iflaviridae* family, including Culex iflavi-like virus 4, Culex picorna-like virus, and Hubei picorna-like viruses 41 and 63 (44). These findings align with reports from Mozambique, Korea, and multiple European countries (45–52). Italian *Iflaviridae* strains closely matched the Swiss strain, except for one sample, which clustered under the Californian references, suggesting that climate changes (e.g., rising temperatures and altered precipitation), along with other factors such as transportation and human movement, could contribute to the circulation of both virus lineages, particularly in bordering countries such as Italy and Switzerland (53). Unlike the study by Birnberg et al. (51), which detected Culex iflavi-like virus 4 only in males, we found it in both sexes, suggesting broader environmental persistence. Regarding Italian samples classified as picorna-like viruses, the phylogenetic tree clustered them under the Hubei picorna-like virus, isolated in Spain or in China.

A great number of ISVs herein identified were reported as unclassified in GenBank Taxa, and among them, a higher incidence was found for Culex pipiens-associated Tunisia virus, which appeared to exclusively infect the *C. pipiens* species, and Wenzhou sobemo-like virus 4, whose presence, in turn, was restricted to *A. albopictus*. The Culex pipiens-associated Tunisia virus showed a high incidence (28.6%) in Tuscany, consistent with its presence across European countries like Switzerland, Belgium, and Sweden (45). Notwithstanding, Italian strains of this virus genus clustered separately from the North African references (Fig. 4), suggesting regional diversification (46–48). Instead, the Wenzhou sobemo-like virus 4 genomes, detected in this study, clustered separately from the Spanish and Chinese reference strains and appeared genetically divergent from those collected in the Ticino region (46). In addition, the presence of both Culex pipiens-associated Tunisia virus and Wenzhou sobemo-like virus 4 in adult mosquitoes reared from larvae deposited in Siena and Grosseto peri-urban locations suggested their vertical transmission and, consequently, the virus environmental persistence, in accordance with the high rate of these virus-positive samples. However, as previously stated, further virological investigations are essential to confirm vertical transmission of both Culex pipiens-associated Tunisia virus and Wenzhou sobemo-like virus 4. Remarkably, Daeseongdong virus *2*, previously reported only in South Korea and Australia (54, 55), was identified in both *Culex* and sandflies, marking its first detection in Old World mosquitoes. Furthermore, a tentative virome of Italian sandflies was provided for the first time, demonstrating the prevalence of viruses within the *Tombusviridae*, *Phenuiviridae,* and *Nodaviridae* families, offering new insights into the viral ecology of Phlebotominae spp.

Virus host specificity often restricts ISVs to some arthropods, but environmental and climatic factors can shape virome composition (56, 57). The geographical distribution of the main identified virus families led to a different distribution of mosquito virome between the two Toscana districts (Siena and Grosseto). The results suggest that viruses' sensitivity to geographical factors could vary among mosquito species, depending on the mosquito’s living habits and the viral long-distance spreading ability. Notably, we observed geographic differences: *Flaviviridae, Nodaviridae, Tombusviridae*, and *Xinmoviridae* were found in Siena samples but not in Grosseto, whereas Picornavirales were exclusive to Grosseto samples, suggesting a strict correlation between the area of mosquito circulation and the diversity of virus species found. However, the disparity in sample numbers between sites (29 from Siena vs 6 from Grosseto) may introduce bias, underlining the need for more balanced future sampling.

No potential human pathogenic viruses were identified, except for the PUNV that was detected in one sample of sandflies collected in Siena province. PUNV was discovered and isolated in 2008 from sandflies in Northern Tunisia and is now classified as a unique member of the Punique phlebovirus species within the Phlebovirus *g*enus in the *Phenuiviridae* family. Although not proven to be pathogenic, seroprevalence of PUNV has been identified in humans in Tunisia, albeit at a low rate compared to the predominant Toscana virus (28). Additionally, several unidentified reads in the samples were associated with non-viral reads (mostly bacteria and other microorganisms); however, they might also correspond to novel, unclassified viruses. Therefore, further characterization is ongoing to determine whether they contain novel viral genomes. Along with viral detection, the metagenomic sequencing approach also detected the presence of non-viral reads in most samples, mainly represented by *Wolbachia* spp., a common obligate intracellular bacteria found in a wide range of invertebrate taxa (31). *Wolbachia* has been documented to provide resistance to the infection in mosquitoes with some viruses, such as DENV and Zika virus, with the ability to manipulate reproduction and reduce the transmission of vector-borne pathogens; hence, it has been suggested as a potential tool for vector-borne disease control (58–60).

In Tuscany, mosquitoes and sandflies harbor a diverse virome with distinct geographical patterns. *A. albopictus* in the Siena district primarily carried Wenzhou sobemo-like virus, Aedes Phasmavirus, and Aedes Anphevirus, while *C. pipiens* in both Siena and Grosseto was associated with Culex pipiens-associated Tunisia virus and Picornavirales. In addition, sandflies in Siena harbored Punique phlebovirus, highlighting species-specific viral diversity and localized differences in virus circulation, although these viruses do not currently represent a threat to human health. However, given the limited sample size of some species, such as sandflies, or geographical sampling, it is likely that this study represents only a limited observation of the overall mosquito virome. Nonetheless, the results provide insight into the ecology and evolution of mosquito-borne viruses and emphasize the necessity of ongoing surveillance for potential threats to public health.

## Data Availability

Representative sequence reads generated in this study have been deposited in the NCBI GenBank database under the following BioProject accession numbers: PRJNA1254928, PRJNA1254978, PRJNA1254989, PRJNA1254991, PRJNA1255030, PRJNA1257162, PRJNA1257269, PRJNA1257127, PRJNA1257270, and PRJNA1277535. The associated BioSamples and raw sequence reads are available in the NCBI Sequence Read Archive (SRA). Complete repository details, including individual accession numbers and metadata, are provided in Table S3.

## SUPPLEMENTAL MATERIAL

Supplemental material spectrum.01867-25-s0001.docx Supplemental table legends.

Table S1 spectrum.01867-25-s0002.xls List of viruses and their corresponding taxa identified through metagenomic analysis of insect pools.

Table S2 spectrum.01867-25-s0003.xls Details of the sequences included in the phylogenetic analysis along with their relative distances and standard errors in comparison to the reference sequences.

Table S3 spectrum.01867-25-s0004.xls Information on the deposited sequences, including BioProject and BioSample identifiers, as well as SRR.

ASM does not own the copyrights to Supplemental Material that may be linked to, or accessed through, an article. The authors have granted ASM a non-exclusive, world-wide license to publish the Supplemental Material files. Please contact the corresponding author directly for reuse.
