# Supplementary material for: Metagenomic sequencing discloses the virome composition of mosquitoes and sandflies from Central-Southern Tuscany, Italy
Source: Microbiol Spectr. 2026 Feb 26;14(4):e01867-25. doi: 10.1128/spectrum.01867-25 (PMC13055373; doi:10.1128/spectrum.01867-25)
Supplement: Supplemental material — Supplemental table legends. [file spectrum.01867-25-s0001.docx]

**Supplementary Table 1**. List of viruses and their corresponding taxa identified through metagenomic analysis of insect pools, including sequencing details.

**Supplementary Table 2.** Details of the sequences included in the phylogenetic analysis, along with their relative distances and standard errors in comparison to the reference sequences.

**Supplementary Table 3**. Information on the deposited sequences, including BioProject and BioSample identifiers, as well as SRA accession numbers (SRR).
